# An mHealth Intervention to Support Caregivers in Delivering Safer Home-Based Care: Protocol for a Participatory Approach and Feasibility Study

**DOI:** 10.3390/nursrep16100374

**Published:** 2026-10-21

**Authors:** Erica Busca, Ines Basso, Melania Stedile, Mattia Bellan, Fabiola De Marchi, Dino Stefano Di Massimo, Giovanna Rodella, Cinzia Rollino, Antonio Scalogna, Rosanna Vaschetto, Erika Bassi, Alberto Dal Molin

**Affiliations:** 1 Department of Translational Medicine, University of Piemonte Orientale, Via Solaroli 17, 28100 Novara, Italy; erica.busca@uniupo.it (E.B.); ines.basso@uniupo.it (I.B.); mattia.bellan@med.uniupo.it (M.B.); fabiola.demarchi@uniupo.it (F.D.M.); rosanna.vaschetto@med.uniupo.it (R.V.); erika.bassi@uniupo.it (E.B.); alberto.dalmolin@uniupo.it (A.D.M.); 2 Local Health Authority of Biella, Via Dei Ponderanesi 2, 13875 Ponderano, Italy; dinostefano.dimassimo@aslbi.piemonte.it (D.S.D.M.); cinzia.rollino@aslbi.piemonte.it (C.R.); 3 SIMNOVA-Simulation Center in Medicine and Health Sciences, University of Piemonte Orientale, Via Bernardino Lanino 1, 28100 Novara, Italy; giovanna.rodella@uniupo.it (G.R.); antonio.scalogna@uniupo.it (A.S.)

**Keywords:** patient safety, caregivers, older adults, digital health, community health nursing

## Abstract

**Background/Objectives:** Caregivers play a central role in ensuring the safety of community-dwelling older adults receiving home-based care, yet gaps remain in ongoing educational support and communication with professionals. This protocol describes a two-phase study to customise an existing platform for use as a mobile health (mHealth) intervention supporting caregivers in delivering safer home-based care and Family and Community Nurses (FCNs) in supporting them, and to evaluate its feasibility, acceptability and usability. **Methods:** Phase 1 will apply a User-Centred Design (UCD) approach, articulated into four subphases, involving caregivers and a multidisciplinary panel of professionals from district-based health and social care services. Needs will be identified and prioritised through focus groups, informing the customisation and testing of the platform for use as an mHealth intervention. Phase 2 will adopt a convergent mixed-methods design within a Local Health Authority in Northern Italy, among a sample of at least 10 caregivers and a similar number of FCNs, over a 3-month data collection period. Acceptability and usability will be assessed as preliminary conditions for the adoption of a safety-oriented digital intervention, through quantitative self-reported measures, system-generated usage data, and semi-structured interviews. **Conclusions:** This study is expected to result in a customised platform for use as an mHealth intervention, together with preliminary evidence on its feasibility, acceptability and usability in supporting safer home-based care. Findings may inform future implementation strategies for digitally supported caregiving.

## 1. Introduction

Home- and community-based care is playing an increasingly important role in healthcare systems globally, reflecting a shift from hospital-focused services toward care delivered in patients’ everyday life context [1]. Several factors drive this transition, including the imperative to contain healthcare costs amid growing demand for care, which is fuelled by an ageing population and the rise in chronic conditions. Additionally, shortages of healthcare professionals and older adults’ preference to remain at home for as long as possible further accelerate this shift [1].

Within this evolving model of care, community-based nursing services play an important role in supporting patients and their caregivers. Family and Community Nurses (FCNs), in particular, contribute to continuity of care by involving patients and caregivers in needs assessment, care planning, and care delivery [2,3]. However, organizational barriers, including fragmented communication and documentation across services and shortages of human and material resources, may constrain their capacity to provide continuous support [4,5].

Alongside professional services, informal caregivers provide essential physical, emotional, and organisational assistance, enabling individuals with illness, disability, or frailty to remain at home [6]. As older adults become increasingly dependent on assistance, caregivers may assume responsibility for a range of care activities, from medication management [7,8] to technical procedures traditionally performed by healthcare professionals [9]. Ensuring the safety of these activities can be particularly challenging in the home setting, where care is delivered in environments not specifically designed for healthcare provision and without continuous direct professional supervision [10,11]. A Canadian chart review reported an adverse event incidence of 10.1% per client-year, 56% of which were judged preventable, most commonly injuries from falls, wound infections, psychosocial problems, and adverse outcomes from medication errors [12]. Similarly, a Swedish record review identified at least one adverse event in 37.7% of home healthcare records, 71.6% of which were assessed as preventable [13].

Addressing these safety risks also requires caregivers to develop the knowledge and practical skills needed to carry out care activities, identify potential risks, and respond to changes in the care recipient’s condition. Key areas of safe home caregiving include medication management, mobility assistance, and fall prevention, as highlighted in recent expert consensus recommendations [14]. Maintaining these competencies requires appropriate education and ongoing professional support. During the initial stages of caregiving, caregivers frequently seek information about the illness, its treatment and prognosis, and guidance on care provision [15]. Healthcare professionals, particularly nurses, play an important role in addressing these needs and preparing caregivers to undertake care-related responsibilities [9]. However, evidence suggests that educational support is often concentrated at the beginning of the caregiving trajectory and becomes more occasional over time, while caregivers continue to develop their knowledge and skills through experience and informal sources [9]. Educational programmes addressing safe home care are also frequently short-term, predominantly delivered face-to-face, and characterised by limited follow-up [16]. Together, these findings highlight the importance of complementing existing professional support with strategies that facilitate continued access to educational resources and guidance as caregiving needs evolve.

Digital health technologies, particularly mobile health (mHealth) applications, may offer opportunities to complement existing professional support by providing caregivers with access to evidence-based educational resources and facilitating communication with healthcare professionals [17,18]. Such tools could support continuity of education and communication beyond scheduled care encounters, while remaining integrated with community-based nursing services. To fulfil this potential, however, digital interventions need to be responsive to caregivers’ needs and compatible with their everyday care activities. A user-centred approach that involves intended users in adapting digital interventions to their needs and contexts of use may contribute to their usability and acceptability [19,20,21,22].

Despite the increasing availability of mHealth applications, important gaps remain in their application to home care safety. Many existing applications primarily support patients’ self-management, whereas those specifically developed for informal caregivers often focus on individual conditions, such as dementia, or on caregivers’ well-being, self-efficacy, and quality of life rather than on the safety of the care they provide [23,24,25]. Digital interventions addressing home care safety from the caregiver’s perspective remain limited and tend to focus on specific safety domains, such as fall prevention, or on short-term training rather than ongoing support for everyday caregiving activities [26,27]. Moreover, caregivers are not consistently involved in user-centred processes aimed at adapting digital interventions to their needs and care contexts [28,29,30].

These gaps highlight the need to explore digital interventions that combine ongoing access to safety-related educational resources with communication between informal caregivers and community healthcare professionals. Involving caregivers and professionals in the customisation of such interventions may help ensure that their content and functionalities reflect users’ needs and are compatible with routine care practices. Before investigating their potential effects on home care safety, however, it is necessary to establish whether these interventions are feasible to implement, acceptable to intended users, and usable within everyday caregiving and professional practice. These considerations provide the rationale for a participatory approach to customising an existing digital platform, followed by a preliminary evaluation of its feasibility, acceptability, and usability within routine community-based care.

## 2. Materials and Methods

### 2.1. Study Objectives

This study aims to adapt an existing digital platform for use in community-based care to support informal caregivers of older adults in promoting safe home care, and to evaluate the feasibility, acceptability, and usability of the resulting mHealth intervention.

The specific objectives are:

1. to identify and prioritise, through structured engagement of caregivers and a multidisciplinary panel of professionals, the home care safety needs that should inform the content and configuration of the mHealth intervention;
2. to assess the feasibility of using the mHealth intervention in routine community care and its usability and acceptability among caregivers and FCNs.

Following the study objectives, the research is organised into two sequential phases, each directly aligned with one of the specific objectives.

### 2.2. Phase 1 User-Centred Design Approach

Phase 1 will focus on the participatory customisation of an existing platform for use as an mHealth intervention and will be conducted using a User-Centred Design (UCD) approach [31,32]. UCD is a structured, iterative methodology that involves end users throughout the customisation process to ensure that the platform aligns with their needs, practices, and context of use. In this protocol, customisation refers to the participatory definition of educational content and the configuration of existing platform functions and interface options, without modification of the platform’s source code. This approach supports the systematic integration of user feedback and the progressive refinement of the platform’s initial configuration. In this study, end users include caregivers and professionals working in district-based health and social care services.

The UCD process will be articulated into four sequential subphases: Subphase A, involving the identification of stakeholders and the establishment of the working groups; Subphase B, dedicated to investigating and prioritising user needs; Subphase C, focused on building the initial configuration of the platform; and Subphase D, centred on testing this configuration.

#### 2.2.1. Setting

The study will be conducted in a Local Health Authority (LHA) in Northern Italy, focusing on the district-level organisation of community health and social care services. In the Italian healthcare system, the district is the primary setting for coordinating and delivering community-based care, including health promotion, chronic care management, and support for caregivers of community-dwelling older adults.

#### 2.2.2. Subphase A

The objective of this subphase is to ensure the involvement of key stakeholders and to create stable working groups that will support the participatory process across all subsequent UCD subphases. To this end, two working groups will be constituted, each composed of 6–8 participants [33].

The first working group will include caregivers of community-dwelling older adults identified by the FCNs of the LHA among the families they follow, through purposive, criterion-based sampling ensuring representation across the LHA’s different areas. Eligible participants will be adults who are the main caregiver of an older adult needing assistance in daily activities because of physical and/or cognitive limitations, are directly involved in at least one safety-relevant care activity (e.g., medication management, mobility assistance, technical procedures), and understand spoken and written Italian. Caregivers may be relatives, friends, or other members of the older adult’s social network, with or without financial compensation.

The second working group will consist of a multidisciplinary panel of professionals working in district-based health and social care services, including FCNs, district physicians, social workers and care managers. Professionals will be selected on the basis of their expertise and role in the care of older adults at home and will be invited to participate through internal institutional networks with the support of the LHA Nursing and Allied Health Professions Directorate.

All participants will receive information about the study aims and procedures and will provide written informed consent prior to participation.

#### 2.2.3. Subphase B

The objective of Subphase B is to identify and prioritise end-user needs and, on this basis, to define the educational content and configuration of the platform. The two working groups constituted in Subphase A will take part in separate focus groups, one with caregivers and one with the multidisciplinary panel of professionals.

The focus groups will follow a semi-structured guide. The first part will explore perceived needs related to home care safety. The second part will address the topics to be covered by the educational/informational content, its format (e.g., text, images, video, short quizzes) and accessibility preferences (e.g., text size, navigation, use of visual cards), within the functions supported by the existing platform.

All discussions will be audio-recorded and analysed independently for each group, to identify categories relating to safety needs, content and accessibility preferences. Categories emerging from the two focus groups will then be compared to identify areas of convergence and divergence. Convergent categories will be discussed in a joint session involving representatives of both groups, moderated by the research team, to discuss their relative priority and to reach a shared decision on divergent ones. Where no shared decision is possible, the research team will document the divergence and the rationale for the final choice.

The prioritised content and platform features will be implemented in the initial configuration of the platform (Subphase C).

#### 2.2.4. Subphase C

Subphase C aims to configure the existing Moodle-based platform, implementing the content and features prioritised in Subphase B and defining how the mHealth intervention will be introduced, used and sustained within the routine workflow of FCNs. The platform supports role-based profiles (caregiver, FCN, administrator), educational content in multiple formats (text, images, video, short quizzes), notifications and reminders, asynchronous messaging between caregiver and FCN, self-assessment checklists, and the assignment of content to individual users. An additional interface package adds configurable interface and accessibility options, including adjustable text size, fonts and colour contrast, a simplified menu and visual cards in place of text-based lists. The platform can also be installed on smartphones as a Progressive Web App, currently available in beta. The resulting configuration will be tested in Subphase D.

#### 2.2.5. Subphase D

Within this subphase, the initial configuration of the platform will be tested to identify usability issues and interaction problems. Testing will be conducted with a small, heterogeneous sample of approximately 5–10 participants. Participants will use the platform for the first time during a single testing session. Feedback will be collected through direct observation and informal conversations. Findings from this subphase will be used to refine the configuration prior to the feasibility study.

### 2.3. Phase 2

The feasibility study conducted in Phase 2 will assess the feasibility, acceptability and usability of the mHealth intervention. Interviews will also explore participants’ perceptions of safety and risk management in relation to their use of the platform.

#### 2.3.1. Study Design

The study will adopt a convergent mixed-methods design, in which quantitative and qualitative data are collected concurrently, analysed separately, and subsequently merged to provide a comprehensive understanding of the feasibility of the mHealth intervention [34]. The integration of quantitative and qualitative findings will support an overall assessment of acceptability and usability in routine community care.

#### 2.3.2. Setting and Participants

The study will be conducted within district-based community health and social care services of the same LHA in Northern Italy where Phase 1 was carried out. Participants in Phase 2 will constitute an independent sample from those involved in Phase 1, in order to avoid potential bias arising from prior familiarity with the platform’s customisation process. The study will take place in routine care settings, such as outpatient and home-based care contexts, in which FCNs provide support to caregivers of community-dwelling older adults.

Participants will include FCNs involved in community-based care and caregivers of community-dwelling older adults. FCNs will be identified, in collaboration with district nurse managers, based on their willingness to participate in the study and to recruit eligible caregivers within their routine care activities. All participating FCNs will receive information about the study and will provide written informed consent prior to participation.

Eligible caregivers will be individuals providing assistance with activities of daily living to community-dwelling older adults, including relatives, friends, or other members of their social network, with or without financial compensation. Caregivers will be eligible according to the criteria adopted in Phase 1 (Section 2.2.2), with the additional requirements of being willing to use the platform and having access to a smartphone with a mobile data connection.

Recruitment will be conducted using purposive, criterion-based sampling, aiming to obtain a heterogeneous sample of caregivers in terms of geographical area and, where possible, age and educational level. FCNs will identify eligible caregivers among those they support, provide information about the study, and collect informed consent for participation. A member of the research team will then introduce caregivers to the main functions of the platform and explain the modalities of interaction with the FCNs.

As this is a feasibility study, a formal sample size calculation is not required [35]. It is therefore estimated that at least 10 caregivers and a similar number of FCNs will be recruited.

#### 2.3.3. Evaluation Domains

In this study, feasibility is addressed at two levels: the feasibility of the study procedures, assessed through the study process data described in Section 2.3.4, and the acceptability and usability of the mHealth intervention. Acceptability and usability are the evaluation domains adopted in this feasibility study and provide the theoretical framework for assessing the mHealth intervention. These domains are examined as preliminary conditions for the implementation of a safety-oriented digital intervention in home-based care.

Acceptability is understood as a multifaceted construct reflecting the extent to which individuals delivering or receiving a healthcare intervention consider it appropriate, based on their cognitive and emotional responses, drawing on the Theoretical Framework of Acceptability (TFA) proposed by Sekhon et al. [36]. According to this framework, acceptability is articulated through components such as affective attitude, perceived burden, ethicality, intervention coherence, opportunity costs, perceived effectiveness, and self-efficacy.

Usability will be examined in accordance with the ISO 9241-11:2018 standard, which defines usability as the degree to which a product can be used by specified users to achieve specified goals with effectiveness, efficiency, and satisfaction within a specified context of use [37].

#### 2.3.4. Data Collection

**Quantitative Data**

Quantitative data will include study process data, self-reported measures collected from caregivers and FCNs, and system-generated platform usage data. Data will be collected at predefined time points, including baseline (T0) and follow-up assessments (T1 and T2), as detailed in Table 1: T1, at one month, was chosen to assess usability once participants have had sufficient exposure to the platform’s main functions; T2, at three months, to observe its use once integrated into routine care.

*Study Process Data*

Study process data assess the feasibility of the study procedures and consist of (a) recruitment rate, the proportion of eligible caregivers approached by FCNs who agree to participate; (b) retention rate, the proportion of enrolled caregivers who complete the T2 assessment, with reasons for withdrawal recorded; and (c) completeness of data collection.

*Self-Reported Data*

Sociodemographic and professional characteristics of FCNs: age, gender, educational level, years of professional experience in community-based settings and digital competence, assessed using a single item asking FCNs to rate their confidence in using digital tools for work-related activities.

Sociodemographic characteristics of caregivers: age, gender, educational level, employment status, marital status, relationship to the care recipient, and annual household income.

Caregiving characteristics: duration of caregiving and average time dedicated to caregiving activities.

Health literacy: health literacy will be assessed using the Italian version of the eHealth Literacy Scale (eHEALS), an eight-item self-report instrument measuring perceived skills in accessing, understanding, appraising, and using electronic health information to address health-related problems. Responses are rated on a 5-point Likert scale ranging from 1 (“strongly disagree”) to 5 (“strongly agree”), with higher scores indicating greater perceived eHealth literacy [38].

Caregiver preparedness: Caregiver preparedness will be assessed using the Caregiver Preparedness Scale (CPS), an eight-item instrument measuring caregivers’ perceived readiness to provide physical and emotional care, manage services and emergencies, cope with stress, and interact with the healthcare system. Items are rated on a 5-point Likert scale ranging from 0 (“not at all prepared”) to 4 (“very well prepared”). Total scores range from 0 to 32, with higher scores indicating greater perceived caregiver preparedness [39].

Mobile Application Rating Scale (uMARS): perceived quality of the platform will be assessed using the user version of the Mobile Application Rating Scale (uMARS). The scale comprises 20 items rated on a 5-point Likert scale ranging from 1 (“inadequate”) to 5 (“excellent”) and organised into four objective dimensions (engagement, functionality, aesthetics, and information quality) and one subjective dimension (subjective quality). In addition, uMARS includes a perceived impact section (six items), which evaluates the potential influence of the platform on users’ knowledge, attitudes, intentions, and health-related behaviours. The overall uMARS score is calculated as the mean of the four objective dimension scores, with higher values indicating higher perceived app quality [40].

Italian version of the mHealth App Usability Questionnaire (Ita-MAUQ—standalone patient use): perceived usability of the platform will be assessed using the Italian version of the mHealth App Usability Questionnaire (Ita-MAUQ), the standalone patient use version. This version is currently the only MAUQ validated in Italian and is designed for applications that are primarily used independently by users. The questionnaire consists of 18 items organised into three subscales: ease of use (5 items), interface and satisfaction (7 items), and usefulness (6 items). Responses are provided on a 7-point Likert scale ranging from 1 (“strongly disagree”) to 7 (“strongly agree”), with higher scores indicating better perceived usability [41].

*System-Generated Platform Usage Data*

System-generated data will be extracted from the platform to describe patterns of use during the study period. These data will include, for example, the number of user accounts created, frequency of access, time spent navigating within the platform, and sections consulted.

*Qualitative Data*

All participants will be invited to complete a single semi-structured interview at T2 time point (Table 1). Interviews will be conducted by a nurse researcher to explore aspects of acceptability and usability that cannot be fully captured through the available quantitative instruments and to gain an in-depth understanding of the experience of using the mHealth intervention in routine care practice (Box 1). Interviews will also explore caregivers’ perceptions of safety and risk in home-based care in relation to their experience with the platform. All interviews will be audio-recorded, and the audio files will constitute the primary data source for qualitative analysis.

Box 1 **Example of questions that will be used in the semi-structured interviews**

**Questions for Caregivers**

1. Can you describe how the platform fits into your everyday caregiving activities, particularly those related to keeping the person you assist safe at home?

*Please share a concrete example.*

2. How do you understand the purpose of the mHealth intervention and how it is meant to support you?

*Were there any aspects that you found unclear or difficult to understand?*

3. Can you describe any ways in which the mHealth intervention has influenced how you sup-port the person you assist?

*In what ways, if any, has the mHealth intervention influenced your confidence in identifying or managing potential risks at home?*

**Questions for FCNs**

1. Can you describe how the platform has been incorporated into your routine professional practice?

*Please provide a concrete example.*

2. How do you perceive the fit between the mHealth intervention and your professional values and approach to working with caregivers and care recipients?

3. How useful do you find the platform in supporting your work with caregivers and in pro-moting continuity of care?

*Please describe whether and how the platform has supported communication, monitoring, or caregiver edu-cation.*

**Table 1:** Data collected and assessment time points.

Caregivers	T0 (Baseline)	T1 (1 Month)	T2 (3 Months)
Recruitment rate	×		
Sociodemographic and caregiving characteristics	×		
eHEALS	×		
CPS	×		
uMARS		×	
Ita-MAUQ—standalone patient use		×	
Platform usage data		×	×
Interviews			×
Retention rate			×
Completeness of data collection	×	×	×
**FCNs**			
Sociodemographic and professional data	×		
uMARS		×	
Platform usage data		×	×
Interviews			×
Completeness of data collection	×	×	×

Legend: CPS—Caregiver Preparedness Scale; eHEALS—eHealth Literacy Scale; FCNs—Family and Community Nurses; Ita-MAUQ—Italian version of the mHealth App Usability Questionnaire; uMARS—User version of the Mobile Application Rating Scale.

#### 2.3.5. Data Analysis

Quantitative data will be analysed descriptively. Baseline sociodemographic data, eHealth literacy and caregiver preparedness will be used to describe the sample and considered when interpreting acceptability and usability findings. Continuous variables will be summarised as means and standard deviations (or medians and interquartile ranges, where appropriate), and categorical variables as frequencies and percentages. System-generated platform usage data (collected at T1 and T2) will be summarised to describe patterns of use over time.

Qualitative data from the semi-structured interviews will be analysed using qualitative content analysis [42]. Two researchers will independently read the transcripts to familiarise themselves with the data. Meaningful units of text will then be identified and coded through a combined deductive–inductive, two-round process. In the first round, a deductive approach will be used, applying categories defined a priori based on the constructs of the theoretical frameworks adopted for acceptability (TFA) and usability. In the second, inductive round, interviews will be re-read to identify new codes and emerging categories not captured in the first round of coding. Final categories will then be grouped into themes based on conceptual similarity and recurrence within the data.

Consistent with the convergent mixed-methods design, quantitative and qualitative data will be analysed separately and subsequently integrated during the interpretation phase [34]. Integration will be organised around the two evaluation domains, acceptability and usability, on which both quantitative and qualitative data provide information. For each domain, quantitative and qualitative findings will be brought together, for example in a joint display, and the research team will state the extent to which they converge, diverge or complement each other. Where divergence emerges, possible explanations will be examined and reported transparently.

### 2.4. Ethical Considerations

The study will be conducted in accordance with the Declaration of Helsinki. The study protocol was approved by the Local Ethics Committee (Comitato Etico Territoriale, CET Interaziendale AOU Maggiore della Carità di Novara) (protocol number CE235/2026).

Data will be managed in compliance with EU Regulation 2016/679 (GDPR) [43]. The platform is hosted by a certified Moodle service provider (ISO/IEC 27001, 27017, 27018) on cloud infrastructure located within the European Union, with encrypted storage, access restricted to authorised users through role-based profiles, and logging of access and activities [44,45,46]. Participants will be identified by a unique alphanumeric code and data will be reported only in aggregate form.

## 3. Discussion

This protocol describes a two-phase study in which caregivers and professionals will be involved in the customisation of an existing Moodle-based platform and the feasibility evaluation of the resulting mHealth intervention. The intervention is intended to support informal caregivers of community-dwelling older adults and FCNs in their contribution to safe home-based care. Existing digital interventions for informal caregivers have often focused on specific conditions or on caregiver-related outcomes, while fewer have addressed the safety of everyday care activities, ongoing professional support, or caregiver involvement in their development [22,23,24,25,26,27,28,29,30].

The first phase represents a central component of the study, as the involvement of two working groups will allow the integration of caregivers’ and professionals’ perspectives, both engaged in community-based care. Both groups will discuss safety needs as well as the content and accessibility settings of the platform. While the multidisciplinary panel will bring complementary professional expertise, from clinical risks such as fall prevention to the coordination of services, the involvement of caregivers is a distinctive feature of this approach: their everyday experience may bring out practical difficulties and safety concerns that professionals alone may not identify.

The study setting is consistent with this approach. The intervention will be implemented within an LHA in Northern Italy, a territory characterised by a high proportion of older adults and increasing needs related to chronic conditions, frailty, and long-term care [47,48]. In this context, FCNs play a central role in ensuring continuity of care through an ongoing process of assessment, support, and follow-up of older adults and their families [49]. This family-centred model of care [50] provides an appropriate context for the digital intervention proposed in the present study aimed at supporting caregivers and FCNs in delivering safer home-based care.

Consistent with the needs that will be identified through this participatory process, the platform will be customised to address both caregivers’ informational and educational needs. By providing access to health-related information, educational resources and a connection with FCNs, the mHealth intervention may help strengthen caregivers’ knowledge, confidence and ability to manage daily care activities, and support them in recognising potential risks and implementing appropriate care practices, although these effects will not be assessed in the present feasibility study. This support may be particularly relevant during the transition from hospital to home, a vulnerable phase in which fragmented information, medication-related problems and difficulties in managing new care responsibilities may increase the risk of adverse events [51].

One notable feature of the second phase lies in the adoption of a convergent mixed-methods design, integrating quantitative and qualitative data to capture the complexity of implementing a digital intervention within community-based care. The use of established frameworks, the TFA and the ISO 9241-11:2018 standard, will guide the assessment of acceptability and usability, respectively, enabling the intervention to be examined not only in terms of performance, but also considering users’ experiences influencing its potential adoption.

Beyond informing the customisation of this specific platform, the study may generate insights on the challenges of introducing digital technologies into real-world community care settings, including the need for adequate user preparation, access to appropriate devices and connectivity, and integration into existing care practices. The feasibility evaluation will provide insights into how caregivers and FCNs engage with the intervention, and which factors may facilitate or hinder its adoption. While implemented within a specific local setting, the resulting model may inform future implementation efforts in other territorial and care contexts—including hospital-to-home transitions, where effective communication and caregiver preparation are similarly critical.

### Limitations

This protocol has some limitations. First, the feasibility study will be conducted within a single LHA in Northern Italy. This may limit the generalisability of the findings to healthcare contexts with different organisational models, resources, and digital health infrastructures. Second, the evaluation will cover a limited period of platform use. It will therefore not fully capture long-term engagement, sustainability, or integration of the intervention into routine community-based care practice. Third, the mHealth intervention is built on an existing platform. This is consistent with methodologies for adapting existing interventions to new contexts, in which user-centred processes guide what is preserved and modified within a bounded solution space [52], but it means that some needs identified through the focus groups may be addressed only in part within the platform’s existing functions. Moreover, Moodle was originally designed as a learning management system, and evidence on its use with family caregivers and older adults is still limited [53,54]. To improve its usability, an additional interface package has been acquired, offering options such as adjustable text size and colour contrast, a simplified menu, visual cards and installation on smartphones as a Progressive Web App. Fourth, the platform may be more readily used by caregivers with greater digital confidence. Greater familiarity with digital tools may increase the likelihood of participation and engagement with the mHealth intervention. To mitigate this risk, caregivers will be provided with a user guide describing the platform’s main functions and supporting its use in everyday caregiving activities.

## Data Availability

No new data were created or analyzed in this study. Data sharing is not applicable to this article.

## Public Involvement Statement

Caregivers of community-dwelling older adults and community-based professionals will be actively involved in the conduct of this study.

## Guidelines and Standards Statement

This protocol was drafted using the SPIRIT 2013 checklist [55], adapted according to the recommendations for reporting protocols of pilot and feasibility studies proposed by Thabane and Lancaster [56].

## Abbreviations

The following abbreviations are used in this manuscript:

CPS	Caregiver Preparedness Scale
eHEALS	eHealth Literacy Scale
FCNs	Family and Community Nurses
Ita-MAUQ	Italian version of the mHealth App Usability Questionnaire
mHealth	mobile health
TFA	Theoretical Framework of Acceptability
UCD	User-Centred Design
uMARS	User version of the Mobile Application Rating Scale
